# The gut microbiota from maintenance hemodialysis patients with sarcopenia influences muscle function in mice

**DOI:** 10.3389/fcimb.2023.1225991

**Published:** 2023-09-12

**Authors:** Jie Tang, Hailin Zhang, Lixia Yin, Qifan Zhou, Huipin Zhang

**Affiliations:** ^1^ Lianyungang Clinical College of Nanjing Medical University, The First People’s Hospital of Lianyungang, Lianyungang, China; ^2^ Department of Hemopurification Center, The Affliated Lianyungang Hospital of Xuzhou Medical University, Lianyungang, China

**Keywords:** maintenance hemodialysis, sarcopenia, gut microbiota, 16S rRNA, metabolism, fecal microbiota transplantation

## Abstract

**Background:**

Sarcopenia is a common complication in patients undergoing maintenance hemodialysis (MHD). Growing evidence suggests a close relationship between the gut microbiota and skeletal muscle. However, research on gut microbiota in patients with sarcopenia undergoing MHD (MS) remains scarce. To bridge this knowledge gap, we aimed to evaluate the pathogenic influence of gut microbiota in the skeletal muscle of patients with MS, to clarify the causal association between gut microbiota and skeletal muscle symptoms in patients with MS and identify the potential mechanisms underlying this causal association.

**Methods:**

Fecal samples were collected from 10 patients with MS and 10 patients without MS (MNS). Bacteria were extracted from these samples for transplantation. Mice (n=42) were randomly divided into three groups and, after antibiotic treatment, fecal microbiota transplantation (FMT) was performed once a day for 3 weeks. Skeletal muscle and fecal samples from the mice were collected for 16S rRNA gene sequencing and for histological, real-time PCR, and metabolomic analyses.

**Results:**

Mice colonized with gut microbiota from MS patients exhibited notable decreases in muscle function and muscle mass, compared with FMT from patients with MNS. Moreover, 16S rRNA sequencing revealed that the colonization of MS gut microbiota reduced the abundance of *Akkermansia* in the mouse intestines. Metabolome analysis revealed that seven metabolic pathways were notably disrupted in mice transplanted with MS microbiota.

**Conclusion:**

This study established a connection between skeletal muscle and the gut microbiota of patients with MS, implying that disruption of the gut microbiota may be a driving factor in the development of skeletal muscle disorders in patients undergoing MHD. This finding lays the foundation for understanding the pathogenesis and potential treatment methods for sarcopenia in patients undergoing MHD.

## 1 Introduction

Maintenance hemodialysis (MHD) is an effective primary therapy for patients with end-stage renal disease (ESRD). However, long-term MHD can result in various complications, including sarcopenia (Eldehni, 2022), which is characterized by the gradual loss of muscle mass and function (Cruz-Jentoft et al., 2010). The prevalence of sarcopenia ranges from 13.2% to 68.0% in patients with MHD (Dong et al., 2019; Matsuzawa et al., 2021), which can reduce the lower limb muscle strength of the patients, impair their balance, and increase the risk of adverse outcomes, such as falls (Hotta et al., 2015; Vanden Wyngaert et al., 2022), consequently adding a significant disease burden (Kim et al., 2019). Therefore, early prevention and appropriate treatment of patients with MHD is essential. However, the pathophysiological mechanisms underlying sarcopenia in MHD (MS) remain complex and have not yet been fully elucidated. Recent studies indicate that gut microbiota may have a crucial role in maintaining skeletal muscle homeostasis (Lahiri et al., 2019; Lv et al., 2021).

Gut microbiota refers to a microbial community in the human body composed of 10–100 trillion microorganisms in the intestinal tract (Liu et al., 2021). The diversity and overall composition of a healthy gut microbiota are essential for maintaining normal homeostasis in the human body (Lin and Zhang, 2017; de Vos et al., 2022). Individuals with low muscle mass exhibit significantly reduced gut microbiota diversity compared with healthy individuals (Grosicki et al., 2018; Han et al., 2022). Furthermore, the composition and structure of the gut microbiota undergo substantial changes (Wang et al., 2022), with certain bacterial taxa declining in proportion to a decline in muscle function (Kang et al., 2021). In addition, short-chain fatty acids (SCFAs), which are beneficial metabolites found in the gut, have been shown to have a significant positive correlation with muscle mass (Lv et al., 2021; Han et al., 2022). Moreover, when the gut microbiota was transplanted between groups of mice, the skeletal muscles of the recipient mice displayed phenotypes similar to those of the donor mice (Fielding et al., 2019; Lahiri et al., 2019). While these findings suggest a strong correlation between gut microbiota and muscle mass/function, only a few studies have been conducted on patients undergoing MHD. Moreover, most existing studies on the gut microbiota in MS are observational and are insufficient to prove a causal relationship between the gut microbiota and muscle phenotype. Thus, this study aimed to use fecal microbiota transplantation (FMT) to explore the impact of gut microbiota from patients with MS on gut metabolic pathways and skeletal muscle of mice. This study establishes a theoretical foundation for the clinical prevention and treatment of patients with MS.

## 2 Materials and methods

### 2.1 Participants

Patients undergoing MHD who received treatment at Lianyungang First People’s Hospital between June and August 2022 were selected for screening. The inclusion criteria were: (1) age ≥18 years old; (2) normal communication ability; and (3) receiving hemodialysis treatment three times a week for at least 3 months. Exclusion criteria were: (1) taking probiotics, antibiotics, proton pump inhibitors in the 3 months prior to the commencement of the study; (2) any presence of inflammatory disease, autoimmune disease, or immunosuppressive treatment; (3) any presence of gastrointestinal diseases; (4) and substantial changes in dietary patterns in the week before sampling; (5) any presence of cirrhosis, blood diseases, malignant tumors, and other serious complications; (6) bio-electrical impedance analysis (BIA) should not be performed; (7) a history of alcoholism; (8) vegetarianism and other dietary habits that have substantial effects on gut microbiota. Twenty patients undergoing MHD were selected as donors for the study. These comprised ten patients with MS and ten patients without MS (MNS), matched for age (58.0 ± 11.2) and sex (female: male= 3:2). Fecal samples were collected from these twenty donors; the demographics of whom are shown in **Table 1**. The Ethics Committee of Lianyungang First Hospital approved this study (NO.KY-20220704001).

**Table 1:** Demographics of fecal donors.

Demographics	MHD with sarcopenia(n=10)	MHD with non-sarcopenia(n=10)	*p* value
Gender (F/M)	6/4	6/4	1
Age (years)	58.60 ± 12.21	57.30 ± 10.81	0.804
HDL(mmol/L)	1.35 ± 0.32	1.17 ± 0.47	0.330
LDL(mmol/L)	2.90 ± 0.72	3.14 ± 0.79	0.499
CHOL(mmol/L)	4.79 ± 0.96	5.02 ± 1.21	0.652
TG (mmol/L)	1.67 ± 0.61	2.80 ± 1.87	0.87

F, female; M, male; HDL, high- density lipoprotein; LDL, low- density lipoprotein; CHOL, cholesterol; TG, triglyceride.

### 2.2 Criteria for sarcopenia

The diagnostic criteria of the Asian Working Group for Sarcopenia were used as follows (Chen et al., 2014; Chen et al., 2020): (1) Muscle mass and skeletal muscle mass index (SMI) were measured using a multi-frequency bio-electrical impedance analyzer (BIA; InBody S10; Biospace, Seoul, Korea), with SMI calculated as the skeletal muscle mass in the four limbs divided by height squared. Values of SMI < 7.0 kg/m² for men and SMI < 5.7 kg/m² for women were defined as low muscle mass; (2) Muscle strength was measured using a grip strength dynamometer, with grip strengths of < 28 kg for men and < 18 kg for women defined as low muscle strength; and (3) Physical performance was assessed by measuring the time required to complete a 6 m walk, with a walking speed < 1.0 m/s defined as low physical performance. Sarcopenia is diagnosed when a patient’s SMI decreases and reaches the range of low muscle mass and is accompanied by decreased grip strength and/or slow walking speed. All staff who conducted these tests underwent the same prior training and assessments to ensure consistency in the measurements.

### 2.3 Preparation of donor fecal fluid

Fecal samples were collected from ten patients with MS and ten patients with MNS in sterile tubes and diluted in phosphate-buffered saline (PBS) to obtain a solution with a concentration of 100 mg/mL. After filtering and removing impurities and large particles by settling, 10% sterile glycerol was added. The solution was then stored in a -80°C freezer for subsequent oral gavage (Wang et al., 2020).

### 2.4 Animals

Male C57BL/6 mice (6–8-week-old; n=42) were placed in a specific pathogen-free (SPF) laboratory at a temperature of 24 ± 1 °C with a humidity of 60 ± 5% for at least one week. Mice were housed in separate cages under artificial light for 12 h on a light-dark cycle. The mice were administered a mixture of metronidazole (100 mg/kg), neomycin (100 mg/kg), and vancomycin (50 mg/kg) twice daily, and penicillin (1 mg/mL) was added to the drinking water for 7 days to establish pseudo-germ-free mouse models (Vuong et al., 2020). After antibiotic treatment, the mice were divided into three groups at random: the first group was treated with a bacterial solution from MS patients (TWS); the second group with a bacterial solution from MNS patients (TWN), and the third group was the control group (CG), with sterile PBS. The mice were orally gavaged with 150 μL of the solution once a day for 3 weeks and were then fed normally for 2–3 weeks (Wang et al., 2020) to allow the microbiota to stabilize (The specific procedure is illustrated in **Figure S1**). Following a three-week period of microbial colonization, gentle pressure was applied to the mice’s abdomen to aid defecation. Fecal samples from the mice were then collected in sterile containers. The Animal Care and Use Committee of Kanion approved all animal experiments (NO. 2022080201).

### 2.5 Behavioral tests

Several behavioral tests were performed to assess the muscular condition in mice. These were as follows:

#### 2.5.1 Four-limb hanging test

The ground of each mouse’s cage was covered with a layer of soft bedding, and an inverted grid was placed 80 cm above this bedding. Mice were positioned on the grid, and the duration until they fell off the grid was documented as a measure of the mice’s muscular strength.

#### 2.5.2 Exhaustive swimming test

Mice were given adaptation training in a swimming tank with a water depth of 30cm two days prior to the experiment. During the actual experiment, the mice had a weight attached to their tail equivalent to 5% of their body weight, and the time from the beginning of swimming until exhaustion was recorded to evaluate their muscle endurance. Mice were considered exhausted when their head remained submerged in water for more than 7 seconds without resurfacing.

#### 2.5.3 Fatigue test

To assess the physical function of the mice, a rotation-bar fatigue instrument (YLS-4C, China) was utilized. The mice underwent two days of adaptation training on a rotating rod set at a constant speed of 30 r/min. During the actual experiment, the mice were placed on a rotating rod set at 40 r/min. The time they remained on the rod was then recorded.

### 2.6 Quantification of gene expression in skeletal muscle

Total RNA was extracted from the muscle tissue using TRIzol reagent (Invitrogen), according to the manufacturer’s protocol. cDNA was synthesized from the total RNA using a reverse transcription kit (Vazyme, Nanjing, China). To measure the expression of muscle atrophy genes (*Murf-1*) and myosin heavy chain genes (*MYHCIIA* and *MYHCIIB*) in the muscle tissue, quantitative PCR was conducted using an ABI7300 real-time PCR system (Applied Biosystems, USA). All primers used are listed in **Table 2**.

**Table 2:** The list of primers sequences.

Gene	Forward Primer	Reverse Primer
Murf-1	AGAAGTCGGGGGTCAGGGGACG	GGTCCATGATCACTTCATGGCGGCACGAGG
MYHCIIA	AAGCGAAGAGTAAGGCTGTC	GTGATTGCTTGCAAAGGAAC
MYHCIIB	ACAAGCTGCGGGTGAAGAGC	CAGGACAGTGACAAAGAACG
Rpl13	CTCATCCTGTTCCCCAGGAA	GGGTGGCCAGCTTAAGTTCTT

### 2.7 Histological assessment of muscle mass

After three weeks of microbial colonization, the gastrocnemius muscle of mice was isolated, fixed with 10% formaldehyde, and dehydrated using ethanol and xylene. This Muscle tissue was then embedded in paraffin and sliced into thin sections. The sections were subsequently processed using deparaffinization, stained with hematoxylin and eosin, dehydrated, cleared, and mounted. These sections were then photographed using a light microscope. From these images, the morphology of the gastrocnemius muscle and the cross-sectional area of the muscle fibers were analyzed.

### 2.8 16S rRNA sequencing

DNA was extracted using the E.Z.N.A.^®^ Soil DNA Kit (Omega Bio-Tek, USA). The concentration and purity of this DNA were measured using a NanoDrop2000 spectrophotometer and its quality was evaluated *via* 1% agarose gel electrophoresis. Following this, PCR amplification was conducted using the primers 338F (5’-ACTCCTACGGGAGGCAGCAG-3’) and 806R (5’-GGACTACHVGGGTWTCTAAT-3’) to target the V3-V4 variable region of bacterial 16S ribosomal RNA. After purifying the resultant PCR products and constructing the library, sequencing was performed using the Illumina HiSeq platform.

### 2.9 Metabolomic profiling of mouse fecal samples

To extract the metabolites, samples were precisely weighed into centrifuge tubes and mixed with a 400 µL solution of methanol:water (4:1, v/v). Following a 30-min incubation at -20 °C to precipitate the proteins, the samples were centrifuged at 13000 ×*g* for 15 minutes at 4 °C. The resulting supernatants were transferred to sample vials for LC-MS/MS analysis *via* a UHPLC-Q Exactive HF-X system (Thermo Fisher Scientific). The column used to separate the samples in this study was ACQUITY UPLC HSS T3.

### 2.10 Bioinformatics analysis

#### 2.10.1 Illumina sequencing data analysis

The original sequences underwent quality control checks using Fastp (v0.20.0) software and valid sequences were obtained using FLASH (v1.2.7) software. The processing steps included filtering out base pairs with quality values below 20. To this end, a 50-bp window was established and, if the average quality value within the window dropped below 20, bases were trimmed from the trailing ends of reads. Following this, reads with a length below 50 bp were discarded, as were reads containing ‘N’ bases. Pairs of reads obtained in a paired-end sequencing strategy were merged into a single sequence based on where they overlap. The minimum required overlap length was set at 10 bp. During the merging process, an allowable maximum mismatch rate of 0.2 within the overlapping region was employed and the sequences not meeting this criterion were removed. To differentiate between samples, sequences were demarcated based on the barcodes and primers located at the sequences’ terminal ends. Adjustments were made to the sequence orientation as required, with zero allowable mismatches for barcodes and a maximum of two allowable mismatches for primers. UPARSE software (v7.1) was used, with a similarity of 97% for the operational sequence of taxonomic unit (OTU) clustering (Edgar, 2013). The chimera was removed in the clustering process to obtain the OTU representative sequence. For taxonomic annotation, each sequence was classified using the RDP classifier (v2.2) accessible at http://rdp.cme.msu.edu/. The Silva 16S rRNA database (v138) was used for alignment, with a threshold alignment set to 70%. α-diversity and β-diversity were used to measure diversity within and between the samples, respectively. The measurement of β-diversity was generated using QIIME V.1.9.1. In addition, linear discriminant analysis effect size (LefSe) was employed to detect significant abundance differences between groups and to identify groups with significant abundance differences.

#### 2.10.2 Data processing of non-targeted metabolomics

Progenesis QI software (Waters Corporation, Milford, USA) was used to preprocess the raw LC/MS data. Principal component analysis (PCA), orthogonal partial least squares discriminant analysis (OPLS-DA), and partial least squares discriminant analysis (PLS-DA) were then conducted on this processed data, using the R software package ropls (v1.6.2). Metabolites with significant differences were selected based on their variable importance in the projection (VIP) derived from the OPLS-DA model and the *p*-value from a Student’s *t*-test. Metabolites with VIP > 1.5 and *p* < 0.05 were considered to be statistically significant. Metabolite enrichment and pathway analysis based on the KEGG database were used to determine the differential pathways between the groups.

### 2.11 Statistical analysis

One-way analysis of variance and independent samples *t*-tests were used for statistical analysis of normally distributed data. Differences between groups were compared using the Mann–Whitney U test for nonparametric tests of two independent samples and the Kruskal–Wallis H test for nonparametric tests of multiple independent samples. The association between variables was analyzed using Spearman correlation analysis. False discovery rate (FDR) was used to correct the results of multiple analyses, and differences with *p* < 0.05 were considered statistically significant. The data were analyzed using SPSS (IBM SPSS statistics v20.0) and visualized using GraphPad Prism v8.0.

## 3 Results

### 3.1 FMT from MS and MNS patients decreases muscle strength and function in mice

**Figure 1:**
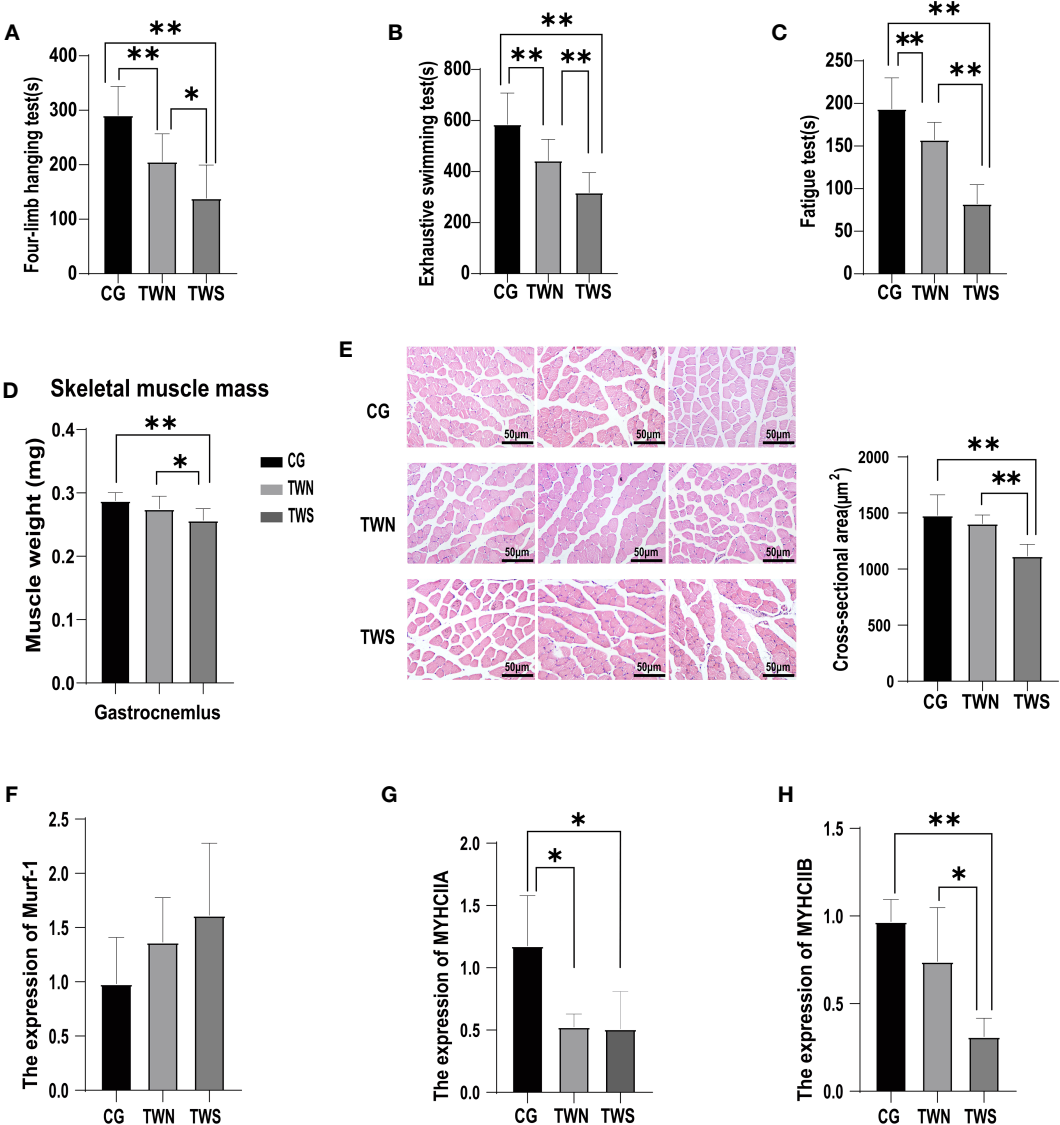
Muscle function and muscle mass in mice colonized with fecal microbiota from sarcopenic and non-sarcopenic maintenance hemodialysis patients. **(A)** Time spent on the four-limb hanging test for the three groups of mice, a proxy for muscle strength (*n*=10); **(B)** Exhaustive swimming time of the three groups of mice, a proxy for muscle endurance (n=14); **(C)** Time spent on the fatigue instrument for the three groups of mice, a proxy for physical function (n=14); **(D)** Weight of gastrocnemius muscle after microflora colonization in the three groups of mice (n=10); **(E)** Three representative images of gastrocnemius muscle sections from CG, TWN, and TWS mice stained for the HE. (*n*=5); **(F–H)** Expression of muscular atrophy gene Murf-1 and myosin heavy chain genes MYHCIIA and MYHCIIB in the tibialis anterior muscle of mice in the three groups (*n*=4). * indicates *p <*0.05, ** indicates *p <*0.01.

Our findings revealed that, compared to in the CG, muscle strength (*p*_MNS_=0.0030; *p*_MS_<0.0001, **Figure 1A**), muscle endurance (*p*_MNS_=0.0006; *p*_MS_<0.0001, **Figure 1B**), and physical function (*p*_MNS_=0.0013; *p*_MS_<0.0001, **Figure 1C**) of mice in the TWS and TWN groups were significantly decreased. Furthermore, the mice in the TWS group experienced more severe damage to muscle strength (*p* =0.0116, **Figure 1A**), muscle endurance (*p* =0.0015, **Figure 1B**), and physical function (*p*<0.0001, **Figure 1C**) than those in the TWN group. These results indicate that the gut microbiota of patients undergoing MHD can affect the health of skeletal muscle in mice, and that the gut microbiota of MS patients can exacerbate the deterioration of muscle symptoms in mice.

### 3.2 FMT from MS patients decreases muscle mass in mice

A significant decrease was observed in the weight of gastrocnemius muscle in the TWS group (*p*_TWS vs CG_ =0.0015, *p*_TWS vs TWN_ =0.0416 **Figure 1D**), indicating the impact of gut microbiota colonization on the muscle mass of the tested mice. To further investigate the changes in muscle mass, we measured the cross-sectional areas of the muscle fibers. The results reveal a significant reduction in the cross-sectional area of the gastrocnemius muscle fibers in the TWS group compared to that in the other two groups (*p*_TWS vs CG_ =0.0024; *p*_TWS vs TWN_ =0.0063, **Figure 1E**). However, the cross-sectional area of muscle fibers showed no significant difference between the TWN and CG groups (*p* =0.3850, **Figure 1E**). *Murf-1* is a crucial gene associated with muscle atrophy in the E3 ubiquitin protease system, which affects skeletal muscle mass by regulating contractile and structural myoglobin (Rom and Reznick, 2016). In this experiment, while the expression of murf-1 was highest in the TWS group, no significant difference was found among the three groups (**Figure 1F**). Additionally, we examined the expression of the myosin heavy chain genes *MYHCIIA* and *MYHCIIB*, which are involved in muscle development and function. The results showed that, while gut microbiota of patients with MNS had reduced expression of *MYHCIIA* (*p*_TWN VS CG_ =0.0194, **Figure 1G**) in the muscle of mice, the effect on *MYHCIIB* was not significant (*p*_TWN VS CG_ =0.1436, **Figure 1H**). Furthermore, the expression of *MYHCIIA* (*p*_TWS VS CG_ =0.0194, **Figure 1G**) and *MYHCIIB* (*p*_TWS VS CG_ =0.0036, **Figure 1H**) in the muscle of mice appeared to have been reduced by FMT from patients with MS, with the TWS group showing the most significant reduction. These findings suggest that the gut microbiota from patients with MS may affect muscle mass of mice by modulating the expression of muscle genes.

### 3.3 Difference in microbiota composition between TWS group and TWN group

**Figure 2:**
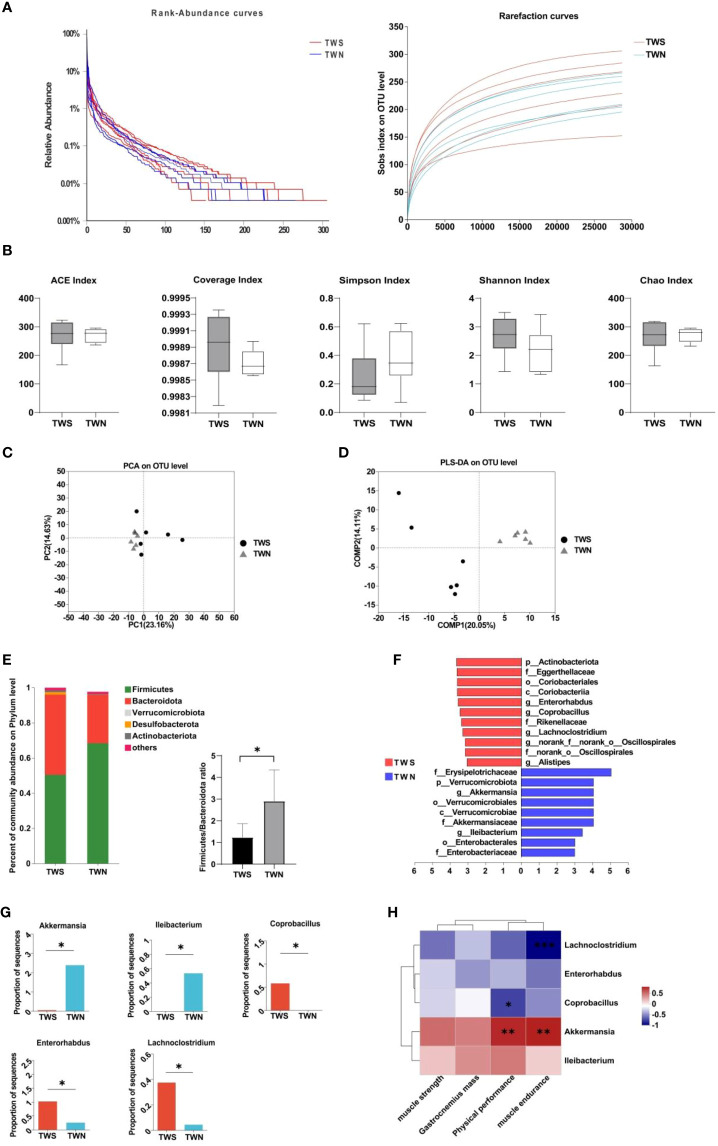
Composition of the fecal microbiota of mice colonized by two different bacterial groups. **(A)** Rank-abundance curve and dilution curve showing microbial diversity in the samples; **(B)** α-diversity measures when comparing TWN with TWS mice; **(C)** PCoA of β diversity in TWS group compared with TWN group; **(D)** PLS-DA of β diversity in TWS group compared with TWN group, R^2 =^ 0.1812, *p* =0.004<0.05; **(E)** Difference of microbiota at phylum level and *Firmicutes*/*Bacteroidete* ratio between the two groups of mice; **(F)** LEfSe analysis of gut microbiota in TWS and TWN groups. (*n*=6, LDA score>3.0); **(G)** Relative abundance of significantly different bacterial genera in the intestinal tract of TWS and TWN mice groups; **(H)** Correlations between the abundance of bacterial genera and muscle function. Positive correlation: red, negative correlation: blue, Spearman correlation analysis was used, * indicates *p <*0.05, ** indicates *p <*0.01, *** indicates *p <*0.001.

First, we generated a rank-abundance curve and used the Sobs index to evaluate the dilution curve and examine the differences in microbial composition between the TWS and TWN group. A gentle curve was generated, indicating ample sequencing samples, and uniform and high-quality data (**Figure 2A**). We then used α-diversity and β-diversity to evaluate the biological composition of gut microbiota in the groups following FMT. Although no significant difference was observed in the α-diversity of gut microbiota between the two groups (*p*_ACE_=0.899; *p*_CHAO_=0.888; *p*_Simpson_=0.293; *p*_Coverage_=0.333; *p*_Shannon_=0.270, **Figure 2B**), significant differences were found in β-diversity (R^2 =^ 0.1812, *p* =0.004, **Figures 2C, D**), suggesting that the composition and structure of gut microbiota were altered after the colonization of flora. Based on this finding, we analyzed the community composition of the gut microbiota in mice and found that the ratio of *Firmicutes*/*Bacteroidetes* at the phylum level in the TWS group was significantly lower compared to in the TWN group (*p* =0.041, **Figure 2E**). To more precisely determine the differences in bacterial composition between the two groups, we used LEfSe analysis combined with LDA values to identify the five genera for which significant differences were observed (**Figures 2F, G**). Among these genera, three were enriched in the gut of TWS mice, including *Enterorhabdus* (*p*=0.045), *Coprobacillus* (*p* =0.028), and *Lachnoclostridium* (*p* =0.031), which belong to *Firmicutes* (2/3) and *Actinobacteria* (1/3), respectively. Two genera were enriched in the gut of the TWN group mice, *Akkermansia* (*p* =0.013) and *Ileibacterium* (*p* =0.028), which belong to *Verrucomicrobiota* and *Firmicutes* (LDA>3), respectively. Spearman’s correlation analysis was then used to investigate the correlation between muscle function and these genera (**Figure 2H**), revealing that abundance of *Akkermansia* was positively correlated with physical performance (*r*=0.775, *p* =0.003) and muscle endurance (*r*=0.796, *p* =0.002), while that of *Lachnoclostridium* was negatively correlated with muscle endurance (*r*= -0.881, *p*<0.01) and of *Coprobacillus* was negatively correlated with physical performance (*r*= -0.661, *p* =0.019).

### 3.4 FMT from MS and MNS patients alters the metabolites in the mice gut

**Figure 3:**
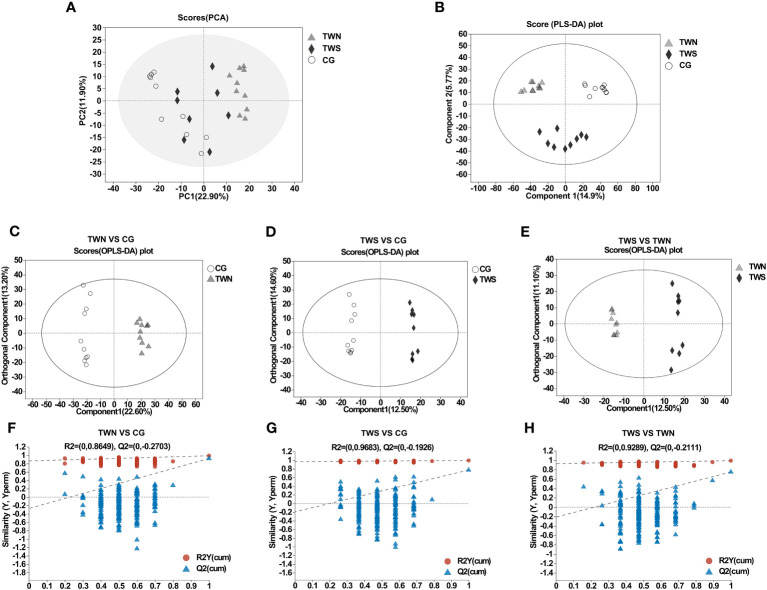
PCA model and OPLS-DA model of gut metabolites in TWS, TWN and CG. **(A)** PCA scores of TWN, TWS and control groups. **(B)** PLS-DA maps of mouse samples in TWN, TWS and control groups. Q^2 =^ 0.806, R^2^Y=0.983, R^2^X=0.377. **(C)** OPLS-DA score plot of TWN (triangle) vs CG mice (circle). Q^2 =^ 0.923, R^2^Y=0.988, R^2^X=0.358. **(D)** OPLS-DA score plot of TWS (diamond) vs CG mice (circle). Q^2 =^ 0.774, R^2^Y=0.995, R^2^X=0.394. **(E)** OPLS-DA score plot of TWS (diamond) vs TWN (triangle). Q^2 =^ 0.751, R^2^Y=0.991, R^2^X=0.237. **(F–H)** validation plot for the model.

Based on the intestinal metabolites of the three groups of mice, we performed PCA and PLS-DA to observe discrete trends among the three groups (**Figures 3A, B**). The PLS-DA analysis of the three groups gave results of Q^2 =^ 0.806, R^2^Y = 0.983, R^2^X = 0.377, indicating good sample aggregation within each group and significant separation between the three groups. OPLS-DA was then performed and this revealed significant separation of metabolites among the three groups (**Figures 3C–E**). Moreover, the parameters of each group demonstrated that the OPLS-DA model established in this study had good predictive ability, enabling an in-depth analysis of differential metabolites (**Figures 3F–H**). Overall, the combined graphical analyses of PCA, PLS-DA, and OPLS-DA show that the gut microbiota of patients with MS and MNS induced changes in the intestinal metabolism of mice. Specific differential metabolites are shown in **Table S1**.

### 3.5 Differential metabolite pathway analysis

**Figure 4:**
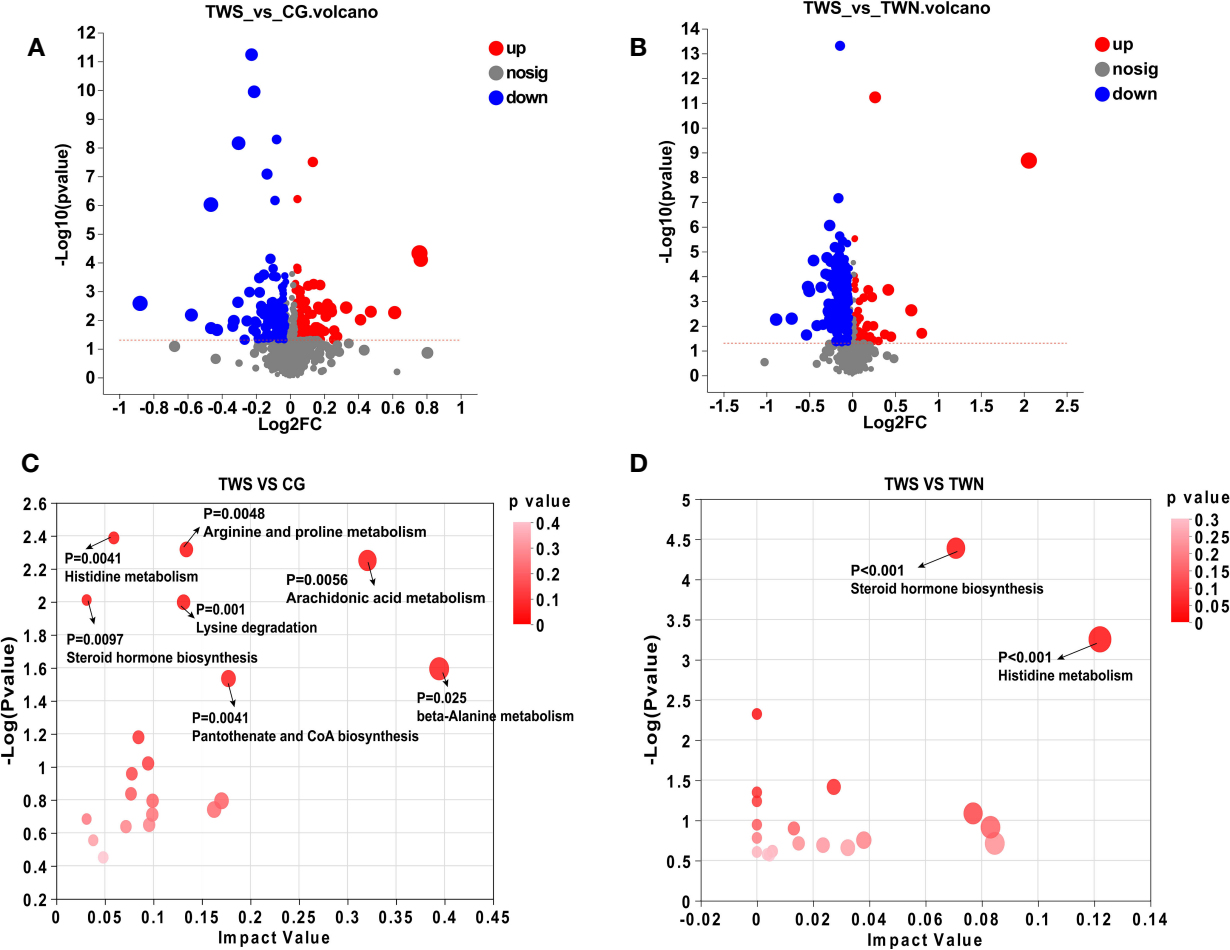
Analysis of differential metabolites and metabolic pathways. **(A)** Volcano plot of different metabolites in the gut of TWS group and CG group; **(B)** Volcano plot of intestinal differential metabolites in TWS and TWN groups; **(C)** Analysis of intestinal differential metabolic pathways between TWS and CG groups; **(D)** Analysis of intestinal differential metabolic pathways in TWS and TWN intervention groups. (*p* < 0.05, VIP value > 1.5).

Based on the metabolic model, differential analysis of metabolites was conducted, and *p* < 0.05 and VIP value > 1.5 were selected for screening. A total of 270 significantly expressed differential metabolites were found between TWS and CG mice, in addition to 225 significantly expressed differential metabolites that were found between TWS and TWN mice (**Figures 4A, B**). Metabolic sets were established for the analyzed differential metabolites, and KEGG pathway enrichment analysis was used to determine the biological pathways involved in the metabolism of differentially expressed metabolites and their biological roles. The results show that seven metabolic pathways were significantly altered between TWS and CG mice (**Figure 4C**), comprising histidine metabolism, steroid hormone biosynthesis, lysine degradation, arachidonic acid metabolism, arginine and proline metabolism, pantothenate and CoA biosynthesis, and β-alanine metabolism. Of these seven pathways, histidine metabolism and β-alanine metabolism appeared to be the two most important metabolic pathways based on the *p*-value and influence value in the KEGG analysis. Two metabolic pathways, histidine metabolism and steroid hormone biosynthesis, differed significantly between TWS and TWN groups (**Figure 4D**). These findings suggest that disruptions to specific metabolic pathways may provide the link between gut microbiota from patients with MS and skeletal muscle symptoms in mice. Abnormalities in histidine and β-alanine metabolism are the main metabolic pathways affecting skeletal muscle health, while further disturbances to histidine metabolism and steroid hormone biosynthesis pathways may be contributing factors to the continuous deterioration of skeletal muscle symptoms.

## 4 Discussion

Our previous research revealed significant differences in the gut microbiota’s structure, composition, and abundance between MNS and MS patients (Zhou et al., 2021). Those results suggested that gut microbiota disorders in patients undergoing MHD may be an important contributing factor to the development of sarcopenia. This finding is in line with the strong correlation between gut microbiota and muscle health established in previous studies (Fielding et al., 2019; Liu et al., 2021). To further investigate the relationship between gut microbiota and skeletal muscle health in patients with MS, we conducted this study to test the hypothesis that gut microbiota colonization in patients with MS could impact the health of skeletal muscles in mice by altering gut metabolic pathways. Our findings support this hypothesis, as evidenced by the results of 16S rRNA and metabolomics sequencing, which showed that the gut microbiota of MS patients can affect the gut microbes, gut metabolism, and ultimately, skeletal muscle health of mice. Taken together, we believe that our study highlights the crucial role of gut microbiota in the development of sarcopenia in patients undergoing MHD and emphasizes the need for further research in this area.

Studies in animal models have shown that FMT can produce a muscle phenotype similar to that of the donor in the recipient animals (Fielding et al., 2019; Lahiri et al., 2019). In this study, we examined muscle function and mass in mice after FMT and found that the muscle phenotype of patients with MS could be transferred to mice *via* transplantation, suggesting that disturbances in the gut microbiota of MS patients play a crucial role in skeletal muscle homeostasis. To investigate the core flora affecting skeletal muscle, we further analyzed the gut microbiota of the two groups of mice that underwent FMT. Our results indicated that structural changes in the gut microbiota of patients with MS are the primary mechanism underlying skeletal muscle symptoms. Previous studies have shown that a higher *Firmicutes*/*Bacteroidetes* ratio in the gut may be a protective indicator of good bodily composition and metabolic status in elderly individuals (Han et al., 2022), while people with low muscle mass have lower F/B ratios (Yamamoto et al., 2022). Consistent with these findings, our study revealed that the F/B ratio of gut microbiota in TWS mice was significantly lower compared to in the TWN mice, indicating that alterations in the abundance of *Firmicutes* and *Bacteroidetes* may contribute to the pathogenesis of skeletal muscle disease.

By assigning LDA values to the microflora, we found an important difference between the levels of the bacterial genus *Akkermansia* in the gut microbiota of the two groups of mice. *Akkermansia* is considered a beneficial genus of bacteria that promotes the anti-inflammatory and antioxidant state of the intestine through the production of short-chain fatty acids and the maintenance of intestinal homeostasis (Kim et al., 2020; Wu et al., 2021). In addition, *Akkermansia* was enriched in the intestines of both athletes with excellent body composition (Clarke et al., 2014) and mice with high muscle function (Zhang et al., 2022). These enriched levels of *Akkermansia* may be related to testosterone metabolism (Zhang et al., 2021). Moreover, studies in mice have shown that *Akkermansia* treatment reduces the expression of muscular atrophy genes in mouse muscles and increases the expression of proliferator-activated receptor gamma coactivator 1 alpha (PGC-1α) (Byeon et al., 2022). This result indicates that *Akkermansia* plays an essential role in maintaining the health of skeletal muscle. In line with these previous studies, this study revealed that *Akkermansia* was significantly decreased in the gut of mice with low muscle function, suggesting that the reduced structural abundance of *Akkermansia* may strongly influence the muscle status of patients with MS. This result suggest that supplementation with *Akkermansia* may improve skeletal muscle symptoms in MS patients.

The metabolic activity of the gut microbiota is crucial for the host, and its metabolites are closely related to health. To elucidate the metabolic mechanism by which the gut microbiota affects skeletal muscle in patients with MS, we analyzed intestinal metabolites in mice using LC-MS. The results reveal that FMT greatly altered normal gut metabolism in mice. In addition, we identified seven significantly altered metabolic pathways in the intestines of mice between the CG and TWS groups, three of which were amino acid metabolism, two were lipid metabolism, and the remaining two were metabolism of other amino acids and metabolism of cofactors and vitamins. Additionally, two distinct metabolic pathways, amino acid and lipid metabolism, were identified in the guts of the TWN and TWS groups. These findings suggest that the impact of the gut microbiota on skeletal muscle status in MS patients may be attributed to changes in amino acid and lipid metabolism.

The metabolism of β-alanine and histidine may be the primary pathways by which gut microbiota affect skeletal muscle abnormalities in patients with MS. β-alanine, a non-protein-derived amino acid (Matthews and Traut, 1987), is a major metabolite in β-alanine metabolism. After production in the liver, β-alanine is absorbed by tissues, such as skeletal muscle, where it combines with histidine to form carnosine (Matthews et al., 2019). Carnosine is one of the most abundant metabolites in mammalian skeletal muscle (Harris et al., 2006) and helps maintain pH homeostasis in muscle cells (Artioli et al., 2010). Studies have shown that β-alanine supplementation may improve athletic performance and muscle strength in the elderly population (Furst et al., 2018), as well as increase carnosine and improve body fat composition in athletes (Carvalho et al., 2018). Moreover, oral administration of β-alanine increases the concentration of muscle carnosine and enhances exercise endurance in patients with chronic obstructive pulmonary disease (Meys et al., 2020; De Brandt et al., 2022). β-Alanine metabolism is strongly correlated with chronic fatigue and physical performance in adolescents (Zhao et al., 2022). Histidine metabolism is another important pathway that can promote lipid accumulation through the H1R signaling pathway (Zhu et al., 2022). High levels of lipids in skeletal muscle can lead to insulin resistance (Al Saedi et al., 2022) and influence muscle mass by reducing the phosphorylation of protein kinase B (Akt) (Bailey et al., 2006), which is closely related to postmenopausal osteoporosis and sarcopenia (Wei et al., 2021). In addition to these metabolic pathways, steroid hormone biosynthesis plays an important role in sarcopenia progression. Steroid hormones (such as estrogen and androgen) are positively correlated with skeletal muscle mass and upper body strength (Bochud et al., 2019), which can promote muscle protein synthesis (Hansen et al., 2012) and affect the anabolism of skeletal muscles (Lowe et al., 2010). Although the exact mechanisms of these metabolic pathways in sarcopenia in patients with MS have not yet been fully elucidated, studies have demonstrated their substantial impact on skeletal muscle. Therefore, we speculate that a complex pathophysiological network may be involved between the gut microbiota and gut metabolism in regulating the healthy development of skeletal muscles in patients with MS. When the gut microbiota and metabolism are disturbed, this network may affect the normal state of skeletal muscle. Alterations in β-alanine and histidine metabolism may explain how the gut microbiota from patients with MS affects skeletal muscle symptoms in mice. Further dysregulation of steroid hormone biosynthesis and histidine metabolism may be the major factors contributing to the worsening of skeletal muscle symptoms.

In conclusion, our study established a causal relationship between gut microbiota from patients with MS and skeletal muscle symptoms in mice, identified several gut metabolic pathway involved in skeletal muscle homeostasis, emphasized the strong role of gut microbiota in skeletal muscle health, and suggested the potential of targeted therapy of gut microbiota to improve muscle symptoms and thereby prevent sarcopenia development. However, our study has several limitations. First, we could not identify the specific pathogenic bacteria causing skeletal muscle lesions in patients with MS, and we only sequenced the two groups of mice with transplanted gut microbiota and not the control group, thus limiting our comparative analysis. Additionally, although we found a close association between the lack of *Akkermansia*, changes in metabolic pathways, and skeletal muscle lesions, the specific molecular mechanisms affecting skeletal muscle require further verification. Therefore, future studies should focus on elucidating the molecular mechanisms underlying the effects of gut microbiota and its metabolites on skeletal muscle. Additionally, such studies should utilize sarcopenia specific model mice for further investigation to develop intestinal-targeted intervention programs for patients with MS and help provide evidence for the clinical treatment of patients with sarcopenia undergoing MHD.

## Data availability statement

The datasets presented in this study can be found in online repositories. The names of the repository/repositories and accession number(s) can be found below: The data presented in the study are deposited in the National Center for Biotechnology Information (NCBI) repository, accession number PRJNA975148.

## Ethics statement

The studies involving humans were approved by The Ethics Committee of Lianyungang First Hospital. The studies were conducted in accordance with the local legislation and institutional requirements. The participants provided their written informed consent to participate in this study. The animal study was approved by The Animal Care and Use Committee of Kanion. The study was conducted in accordance with the local legislation and institutional requirements.

## Author contributions

JT and HaZ jointly designed the study. JT, HaZ, LY, QZ, HuZ conducted data collection and experiments. JT wrote the manuscript, and HaZ edited the manuscript. All authors contributed to the article and approved the submitted version.
