# Supplementary figures and images for: The gut microbiota from maintenance hemodialysis patients with sarcopenia influences muscle function in mice
Source: Front Cell Infect Microbiol. 2023 Sep 12;13:1225991. doi: 10.3389/fcimb.2023.1225991 (PMC10523162; doi:10.3389/fcimb.2023.1225991)

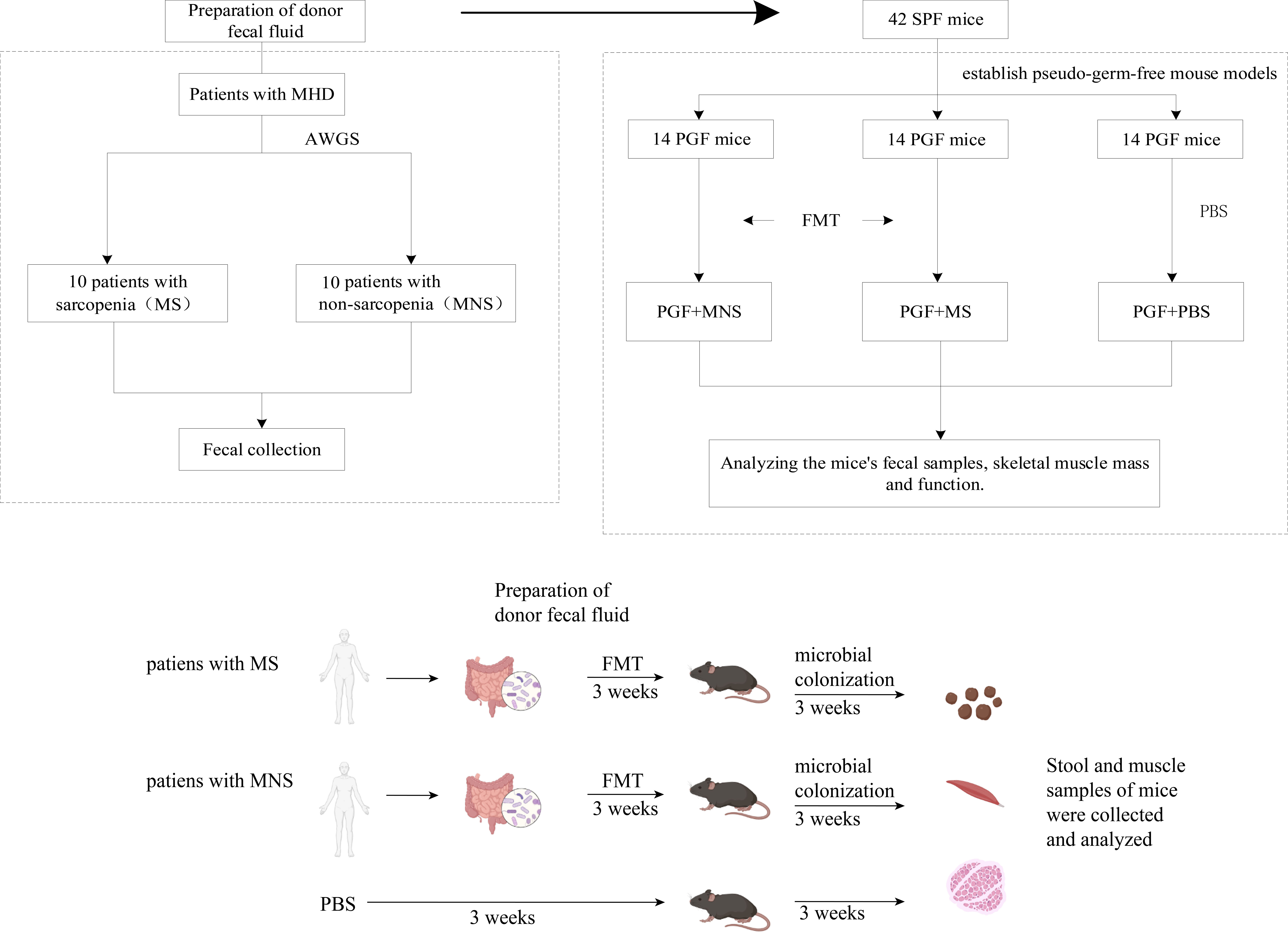

Supplement: Supplementary file 1 [file Image_1.jpeg]
